# In Vitro Evaluation of Manual Torque Values Applied to Implant-Abutment Complex by Different Clinicians and Abutment Screw Loosening

**DOI:** 10.1155/2017/7376261

**Published:** 2017-04-03

**Authors:** Onur Dincer Kose, Burcin Karataslı, Sabit Demircan, Taha Emre Kose, Erhan Cene, Serhan Aydın Aya, Mehmet Ali Erdem, Abdulkadir Burak Cankaya

**Affiliations:** ^1^Private Practice, Istanbul, Turkey; ^2^Department of Prosthodontics, Faculty of Dentistry, Istanbul University, Istanbul, Turkey; ^3^Department of Dentomaxillofacial Radiology, Faculty of Dentistry, Istanbul University, Istanbul, Turkey; ^4^Department of Statistics, Yıldız Technical University, Istanbul, Turkey; ^5^Department of Mechanical Engineering, Faculty of Mechanical Engineering, Istanbul Technical University, Istanbul, Turkey; ^6^Department of Oral and Maxillofacial Surgery, Faculty of Dentistry, Istanbul University, Istanbul, Turkey

## Abstract

Preload is applied to screws manually or using a torque wrench in dental implant systems, and the preload applied must be appropriate for the purpose. The aim of this study was to assess screw loosening and bending/torsional moments applied by clinicians of various specialties following application of manual tightening torque to combinations of implants and abutments. Ten-millimeter implants of 3.7 and 4.1 mm diameters and standard or solid abutments were used. Each group contained five implant-abutment combinations. The control and experimental groups comprised 20 and 160 specimens, respectively. Implants in the experimental group were tightened by dentists of different specialties. Torsional and bending moments during tightening were measured using a strain gauge. Control group and implants with preload values close to the ideal preload were subjected to a dynamic loading test at 150 N, 15 Hz, and 85,000 cycles. The implants that deformed in this test were examined using an optical microscope to assess deformities. Manual tightening did not yield the manufacturer-recommended preload values. Dynamic loading testing suggested early screw loosening/fracture in samples with insufficient preload.

## 1. Introduction

Dental implants offer extensive treatment options for patients who are completely or partially edentulous [1]. Osseointegrated dental implants are the goal in clinical practice; however, there may be complications such as screw loosening and screw, implant, or denture fractures [1–3].

Preload applied by rotational movement of abutment screws is essential for retention in systems in which the connection between the implant and abutment is maintained via a screw. When tightening torque is applied to abutment screws, the screws function as a hard spring. The elastic recovery feature of abutment screws creates a connection force that keeps components together [4]. The preload forms a compressive force between the head of abutment screws and the abutment platform, the abutment and implant, and the abutment and the implant interior grooves, and so holds these components together [5].

The preload primarily depends on the applied torque force and secondarily on the material, the design of the screw head and grooves, and the surface roughness between the implant and abutment [5]. The microroughness of the implant components is a major determinant of the preload. The application of tightening torque flattens the irregularities on the surface of components. The energy required for this flattening causes the final load force to decrease; the lost energy goes to frictional resistance.

For clinical success, screw retention should be stable and constant. For this reason, the magnitude of the preload force is critical. The magnitude of torque applied for the preload depends on the yield strength of the screw and the strength of the bone-implant interface [5].

Use of the optimum tightening torque for implant-abutment complex is vital for clinical success. The risk of loosening is particularly high in abutment screws that are tightened with forces lower than the recommended tightening torque. However, tightening an abutment screw with high forces causes its yield strength to be exceeded and the screw to lose its mechanical characteristics due to plastic deformation [6].

Loosening or fracture of abutment screws is one of the most common mechanical complications [2]. The loosening of abutment or prosthesis screws is more likely to be encountered in single-tooth implants and in the presence of parafunction and cantilevers [6, 7]. The screw loosening rate after 5 years has been reported to be 5.8–12.7% [8, 9].

Screw loosening can be caused by inadequate tightening torque, excess mechanical loads, inconsistencies in the material and design of the abutment screw, vibration during functional loading, temperature changes in the oral cavity, and misplacement of the implant [2, 6, 10].

Screw loosening can cause implant and screw fracture, unbalanced distribution of occlusal forces, a microgap space between the implant and abutment that can allow bacteria in by causing a micromovement, peri-implant inflammation, and loss of osseointegration due to the microgap [4, 6, 10].

Bickford defined screw loosening as a two-stage process. Initially, external functional forces affect the screw connection and consequently cause the tightening torque to diminish. Vibration and micromovement loosen the screw and decrease the effective preload force. Secondly, the preload force decreases below the critical level, which causes the grooves to rotate and loss of function in the screw joint [11]. After screw loosening, metal fatigue causes screw fracture [12].

The antirotational element and preload of the screw joint are important in preventing screw loosening in implant abutments [5]. After the first tightening of the abutment screw using a torque wrench, the same process should be repeated to generate the desired preload force due to the loosening that occurs after the initial tightening [7].

Although some studies have focused on tightening torque, namely, the preload applied to the implant-abutment screw using a ratchet torque wrench, and behaviors during dynamic loading testing, few have evaluated manual application of tightening torque to screws used frequently for implant-supported prostheses. In particular, not many studies have analyzed the bending and torsional moments that occur during manual application of tightening torque by physicians of various specialties and both genders. Therefore, we aimed to analyze the right-left, front-back bending moment and torsional moment during manual application of tightening torque to screws by female and male physicians of different specialties and evaluated the rates of early complications.

## 2. Materials and Methods

Implant KA (Mode Medical, Istanbul, Turkey) bone-level implants with platform switching were used, which included Morse conical connections and Oktafiks conical connections that start as the conical connections finish. A total of 90 (10 mm height, 3.7 mm diameter) and 90 (4.1 mm diameter) bone-level implants were used. We also used standard and solid abutments with 2 mm gingival height, which were compatible with the implants.

**Figure 1:**
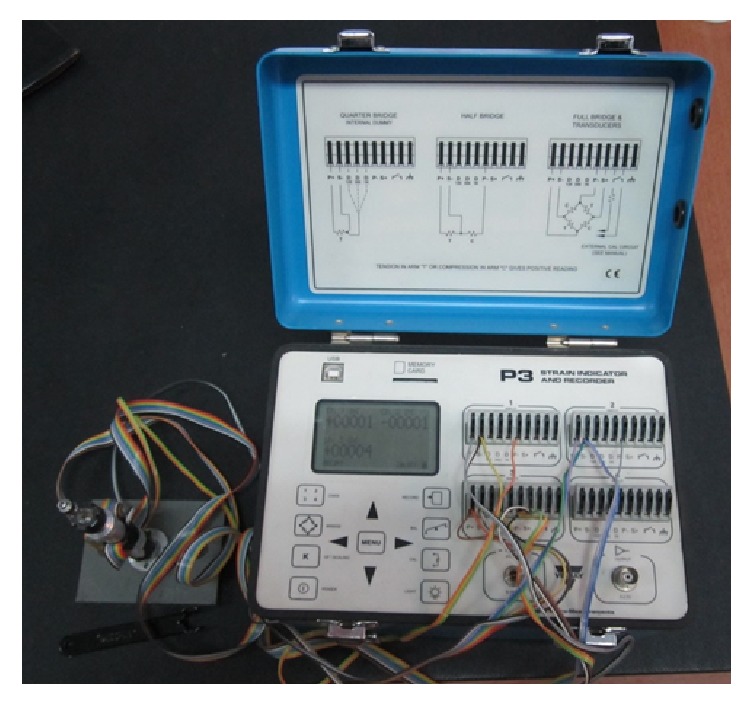
Experimental set-up.

Each specimen consisted of an implant, abutment, abutment screw, manual screwdriver, apparatus on which the implant complex was held using CNC pliers, a strain gauge connected to the strain indicator on which the components were stabilized, and a strain gauge (Figure 1). Twenty implants (five implants of each diameter and abutment type) comprised the control group and were tightened using the torque value recommended by the manufacturer, 25 Ncm, using the manufacturer-supplied, calibrated ratchet wrench. The remaining 160 implants were categorized into four groups according to implant diameter and abutment type. Manual tightening of the abutment screw in the experimental group was performed by dentists with implantology experience who were oral and maxillofacial surgeons, prosthodontists, and periodontists and those of no specialty who had at least 5 years of experience. The physicians were divided into male and female groups.

**Figure 2:**
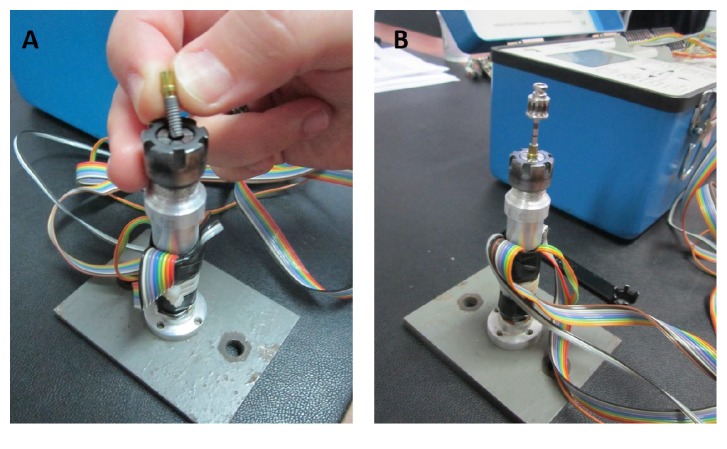
(A) Implant placement in the experimental set-up. (B) Handheld screwdriver placement in the experimental set-up.

The physicians applied torque to the abutment screw using a manual screwdriver before cementation of implant-supported prostheses. The participants were blinded to the implant diameter and abutment type at the time of application of manual tightening torque. The largest torsional moment that occurred during tightening torque application manually or using a ratchet torque wrench was measured. The torque values and bending moments in the right-left or front-back directions during application of tightening torque to the abutment screw were measured using a digital strain gauge indicator (VISHAY P3 Strain Indicator and Recorder, Wendell, NC, USA) (Figure 2).

The experiments were recorded using a digital video camera, and the largest torsional and bending moments were determined. This method reduces the error caused by rapid changes in the moment values.

Twenty manually tightened samples from the experimental group that had close to the ideal torque value (5 of each implant diameter and abutment type) and 20 samples from the control group were subjected to dynamic loading testing using a servohydraulic testing machine in accordance with ISO 14801. The parameters 150 N, 15 Hz, and 85,000 cycles, which represent a 1-month mastication cycle and 25°C (room temperature), were used. To imitate the crestal bone loss in endosteal implants, implants attached to plier from 3 mm apically from the implant-abutment connection. A force of 150 N was applied to the test sample, which was placed on a 30° slope at 15 Hz, 2 mm from the abutment center. The tip of the mechanical set-up moved vertically in 5 mm steps until it contacted the inclined surface of the test sample and then slid 2 mm in a lateral direction.

**Figure 3:**
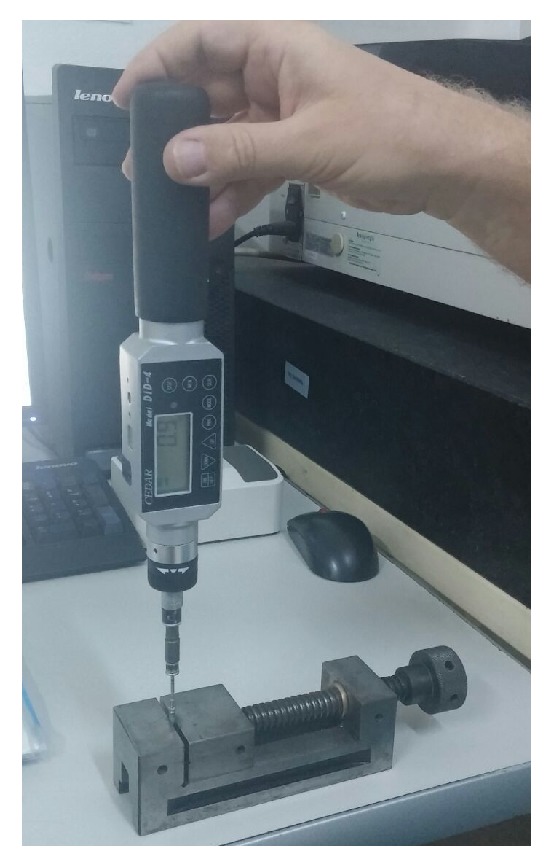
Measurement of loosening torque using a torque tester.

Samples that showed no macroscopic deformation after the dynamic fatigue test were checked for deformation and fractures using an optical microscope (Vision Measuring Machine Mitutoyo 359, Quick Scope; Mitutoyo, Kawasaki, Japan). The loosening torque value was determined using a torquemeter (Torque Tester; Crane Electronics Inc., Hinckley, UK) (Figure 3).

This study did not involve prosthetic restorations due to the difficulty in maintaining standardization and for the elimination of related effective parameters.

Differences in bending and torsional moments according to gender were evaluated by *t*-test, and differences according to specialty were subjected to one-way analysis of variance (ANOVA). The reasons for the results found by ANOVA were investigated using the Bonferroni test. The analyses were performed using a 95% confidence interval and significance level of 0.05. The relationship between the initial tightening torque values and the final state of the implant-abutment screw complex was evaluated by calculating Spearman's Rho correlation coefficient and by performing ANOVA.

The right-left bending of the implant-abutment complex during application of manual torque is referred to as the Ch1 bending moment. The front-back bending is known as the Ch2 bending moment, and the torque force applied is termed the Ch3 torsional moment.

The Ch3 torsional moment values in the control group were those recommended by the manufacturer. Therefore, the Ch3 torsional moment values of the control group were included in the statistical evaluation for comparison.

## 3. Results

### 3.1. Analyses of Bending and Torsional Moments according to Specialty

The mean Ch1, Ch2, and Ch3 moments according to specialty are shown in Table 3. Prosthodontists and dentists showed the highest and lowest Ch1 moment values, respectively. Similarly, oral and maxillofacial surgeons and dentists showed the highest and lowest Ch2 moment values, respectively. In addition, none of the specialties achieved a torsional Ch3 moment of 25 Ncm, which is recommended by the manufacturer. The prosthodontists had the highest Ch3 moment (10.989 Ncm), which is considerably lower than the recommended value.

The differences in Ch1 and Ch2 bending moments and Ch3 torsional moments among specialties were investigated with ANOVA and Bonferroni tests. The Ch2 bending moment (*p* = 0.002) and the Ch3 torsional moment (*p* < 0.001), but not the Ch1 bending moment (*p* = 0.067), were statistically significant (Table 1). Prosthodontists and dentists exhibited the statistically significant difference in Ch1 values (*p* = 0.035), and oral and maxillofacial surgeons and dentists showed statistically significant difference in Ch2 values (*p* = 0.025). The control group exhibited a significant difference in Ch3 values compared to subjects of all specialties (*p* < 0.001) (Table 2).

### 3.2. Analyses of Bending and Torsional Moments by Gender

Males applied higher Ch1 and Ch2 bending moments on the abutment screws than females (26.737 versus 19.718 Ncm and 19.567 versus 15.693 Ncm, respectively) (Table 7). However, the latter difference was not significant (Table 4). The control group exceeded the recommended Ch3 torsional moment value of 25 Ncm (25.988 Ncm). However, both the male group (10.295 Ncm) and the female group (8.199 Ncm) failed to achieve the target value.

Females and males exhibited a significant difference in Ch1 bending moment (*p* = 0.021), but not Ch2 bending moment (*p* = 0.137) (Table 4). There was a significant difference between the genders (*p* < 0.001; Table 5), and among the male, female, and control groups (*p* = 0.006 for males–females, *p* < 0.001 for control–males and for control–females) (Table 6).

### 3.3. Experimental Group Results

Of the experimental group samples subjected to dynamic fatigue testing (*n* = 20), 9 (45%) had screw fractures and 8 (40%) had an opening on the pressured side. Furthermore, 3 (15%) showed only loosening, not deformation. The mean initial tightening torque values of the group with fractures, an opening on the pressured side, and loosening were 13.79, 12.85, and 16.34 Ncm, respectively.

Correlation test results showed that there was no relationship between the initial tightening torque, namely, the preload force, and the occurrence of a fracture/opening on the pressured side (Spearman's Rho = −0.001) (Table 8).

In the group that experienced opening due to pressure, the mean loosening torque increased by 15% compared to the initial tightening torque value. In contrast, the mean loosening torque decreased by 34% in the group with only loosening (Table 9).

### 3.4. Control Group Results

The mean loosening torque value of the control group with 3.7 mm diameter implants and standard abutments decreased by 29%, compared to 34% in the group with 3.7 mm diameter implants and solid abutments.

The mean loosening torque value of the control group samples that were exposed to dynamic fatigue decreased 22% in the group with 4.1 mm diameter implants and standard abutment and 26% in the group with 4.1 mm diameter implants and solid abutment.

The dynamic fatigue test results for the control groups are shown in the right column of Table 9.

## 4. Discussion

Although the preload force used on abutments of various types, diameters, and connection types using a ratchet wrench and the mechanical resistance of the implant-abutment complex have been reported, few studies have focused on manual tightening of abutment screws, which is generally preferred by dentists [13–15].

Application of the optimum torsional moment to the implant-abutment complex is critical for long-term successful prosthetic implant restoration. The implant-abutment connection loosens over time, resulting in microgaps, bacterial colonization, and peri-implantitis. Over time, microgaps progress to macrogaps. In this situation, the surface connection between the implant and abutment is lost, leading to abnormally directed forces on the screw. These phenomena cause complications such as inflammation/infection of the soft tissues and fracture of the screw. To prevent this, it is crucial to apply the optimum torsional force to the implant-abutment connection, ideally using a torque-calibrated ratchet wrench [4, 6, 10]. Screws can be tightened manually if a ratchet wrench is not available.

We evaluated the preload (torque force/torsional moment) on abutment screws depending on specialty and gender, as this can affect implant dental practice. We also assessed the condition of the implants after manual application of a preload force under dynamic loading conditions.

A 30° loading angle, which is close to the tubercle slope of posterior teeth, is also recommended in the ISO 14801 protocol, which was developed for evaluating the mechanical resistance of dental implant materials. In this protocol, the experimental conditions were defined to mimic oral-cavity conditions under dynamic loading conditions. In studies by Steinebrunner et al. (2005), Balfour and O'Brien (1995), and Steinebrunner et al. (2008), a 30° loading angle was used in dynamic loading tests [16–18]. Therefore, we used a loading angle of 30° for the dynamic fatigue test.

It is specified in ISO 14801 that, during dynamic load testing, the 3 mm crestal part of the implant within the material should be excluded to imitate the worst clinical condition and possible crestal bone loss. In the studies by Khraisat et al. (2004), Balfour and O'Brien (1995), and Tsuge and Hagiwara (2009), 3 mm crestal portions of implants were stabilized outside the complex to simulate possible crestal bone loss [5, 17, 19]. Therefore, in this study, dynamic load testing was performed with the samples in the apparatus with the 3 mm neck protruding.

Abutment screws tightened to 30 Ncm for up to 5 × 10^6^ cycles have been reported not to experience complications; however, abutment screws tightened to 20 Ncm experienced complications at 357.162 cycles [20]. Thus, abutment screws with a 20 Ncm preload force can function for 2-3 months and those with a 30 Ncm preload force for 2-3 years without complications [21]. These predictions are based on an individual performing three 15-minute chewing episodes of 60 cycles per minute (1 Hz) daily. Thus, an individual is considered to perform 2,700 chewing cycles per day, and 1,000,000 per year [21, 22]. Therefore, we simulated 1 month of chewing (1,000,000 cycles/12 months) because complications in the implant-abutment complex would probably occur before this time point due to manual tightening of the abutment screw and the low preload force. Therefore, the samples were subjected to a dynamic loading test at 150 N, 15 Hz, and 85,000 cycles at 25°C (room temperature). Also in the ISO 14801 protocol, 2 × 10^6^ cycles are recommended at 2 Hz, while lower speeds and 5 × 10^6^ cycles are advised for velocities of 2–15 Hz.

We used 150 N loading in the dynamic loading test based on the work of Shin et al., who performed a dynamic fatigue test at 10–150 N, 10 Hz, and 10^5^ cycles [10]. Kim et al. conducted a dynamic loading test at 150 N, 6 Hz, and 1 × 10^6^ cycles [23].

The Ch1 bending moment, that is, the right-left inclination of the implant-abutment screw during manual application of tightening torque, applied by prosthodontists was greater than that applied by dentists. Moreover, males applied a greater right-left bending moment than females. Similarly, the Ch2 bending moment, that is, the front-back inclination of the screw, differed between the oral and maxillofacial surgeons and dentists. No study of this phenomenon has to our knowledge been published. We believe that a possible reason for this difference is the application of greater torsional moment by prosthodontists and oral and maxillofacial surgeons to the abutment screw compared with dentists. The reason may be the high probability of the physician sliding towards the lateral forces while applying more torsional moment to the screw.

The difference in bending moments during manual tightening of implant screws would probably not affect an osseointegrated implant. However, in patients with loose trabeculation, these lateral forces may affect the primer stability of the implant during surgery.

In a study by Goheen et al. of 5 oral and maxillofacial surgeons and 11 prosthodontists, the physicians were asked to apply 10, 20, and 32 Ncm torque forces to implants manufactured by Brånemark (Gothenburg, Sweden) using manual wrenches of the same brand. Oral and maxillofacial surgeons applied 23–48% lower torque values with target torque values of 10 and 20 Ncm, respectively. The prosthodontists showed a margin of error of 15% with these target torque values. Therefore, oral and maxillofacial surgeons apply less manual force to abutment screws. Goheen et al. emphasized the importance of standardizing torque applied to abutment screws using a torque device to prevent screw loosening [13]. In our study, the torsional moment and tightening torque values in implant-abutment screws after manual application of tightening torque were significantly lower in all specialties and both genders than in the control group. Therefore, torque values generated by manual tightening were lower than those achieved using a ratchet torque wrench [2, 7]. The prosthetic dentistry specialists applied statistically significantly higher tightening torque than dentists (*p* = 0.001). This was probably because prosthodontists are more experienced in implant superstructures and possibly also abutments and abutment screw systems compared with those of other specialties. Similarly, the fact that nonspecialist dentists showed the lowest values was due to their lack of knowledge and experience with clinical osseointegration and implant-supported prosthetic components. The male participants applied significantly greater tightening torque than the female participants.

Gross et al. reported habitual tightening torque values of 7–14.6 Ncm (manufacturer-recommended value 29–55%); the mean maximum tightening torque values were 9.4–19.9 Ncm (manufacturer-recommended value, 32–79%). Therefore, manual application of tightening yielded torque values lower than that recommended by the manufacturer, suggesting that mechanical torque devices are required [15]. The manual tightening torques in this study were also lower than the recommended values.

In a study by Bousquet et al., 30 lecturers at universities that provided dentistry education (12 females, 18 males) and 24 students in their final year of undergraduate education (10 females, 14 males) manually applied the highest tightening torque possible to implant healing abutments in the right and left first molar tooth area and lower left first incisor tooth area. <10, 10–15, 15–20, and 20–25 Ncm tightening torque groups were formed. There were no significant differences between the areas or between the female and male physicians. However, the lecturers applied a significantly greater tightening torque than the students (*p* = 0.01). Therefore, professional experience influenced torque values. However, 90.8% of the participants applied <15 Ncm to the healing abutments, and 8% applied 15–20 Ncm [14]. In our study, the mean values of all dentists with and without specialties were <15 Ncm (Table 3). Additionally, there was a significant difference in the torsional moment values of the prosthodontist and dentist groups (*p* = 0.001), probably because the prosthodontist group had more experience in dental implant prostheses than the dentists. This agrees with the report by Bousquet et al. describing that professional experience influences tightening torque values.

Quek et al. applied torque forces of 20% lower than the recommended torque value (16 Ncm), at the recommended torque value (20 Ncm), and 20% greater than the recommended torque value (24 Ncm) to abutments of implants of diameters 3.3, 3.75, and 5 mm and performed dynamic loading testing. The three torque values differed significantly among the implant diameter groups; however, abutment screw and implant fractures occurred in the 3.3 mm diameter implants. The number of complications due to mechanical fatigue under load is higher in implants of lesser diameter [24]. Quek et al. found no significant difference between the different torque values and torqued abutments after dynamic loading test, whereas there were complications within an early period in samples with low tightening torque values in our study. This could be because the mean torque values in our study were lower than the lowest torque value applied by Quek et al.

In a study by Xia et al., 30 screws of implant-abutment combinations were tightened to 24, 30 (recommended), and 36 Ncm. Dynamic fatigue testing was performed at 30–300 N, 15 Hz, and 5 × 10^6^ cycles. The low tightening torque values negatively affected the screw joints, and the dynamic loading led to loss of preload force [6]. Similarly, in our study, screw fractures and deformations in the implant and screw joints occurred following application of a low preload force. However, the loosening torque values were higher based on the applied tightening forces in the experimental group samples with openings on the pressured side, which was probably related to deformation-related screw tightening. Although no relationship was found between failure and low preload force, fractures, deformation, and screw loosening were detected. The loading number used in this study was selected to imitate a 1-month chewing cycle. As the failures occurred <1 month after implant loading, the insufficient preload force probably resulted in early complications.

The preload force values manually applied to dental implant-abutment screws did not reach those recommended by the manufacturer. This negatively influenced the continuity of the screw connection against dynamic loads, and so manual application of preload force is not sufficient for clinical success; therefore, the calibrated ratchet torque wrench provided by the manufacturer should be used.

## Figures and Tables

**Table 1:** ANOVA for bending and torsional moments according to specialty.

Moment type	*F*	Sig.
Bending moment (Ch1)	2.230	0.067
Bending moment (Ch2)	4.389	0.002^*∗*^
Torsional moment (Ch3)	134.490	<0.001^*∗*^

*∗* refers to <0.05.

**Table 2:** Bonferroni test for bending and torsional moments according to specialty.

Moment type	(*I*) Specialty	(*J*) Specialty	Mean difference (*I* − *J*) (Ncm)	Std. error	Sig.
Bending moment (Ch1)	Oral and maxillofacial surgeon	Prosthodontist	−6.552	4.245	0.748
		Periodontist	−0.207	4.245	1.000
		Dentist	5.322	4.245	1.000
				
	Prosthodontist	Oral and maxillofacial surgeon	6.552	4.245	0.748
		Periodontist	6.345	4.245	0.822
		Dentist	11.874^*∗*^	4.245	0.035^*∗*^
				
	Periodontist	Oral and maxillofacial surgeon	0.207	4.245	1.000
		Prosthodontist	−6.345	4.245	0.822
		Dentist	5.529	4.245	1.000
				
	Dentist	Oral and maxillofacial surgeon	−5.322	4.245	1.000
		Prosthodontist	−11.874^*∗*^	4.245	0.035^*∗*^
		Periodontist	−5.529	4.245	1.000

Bending moment (Ch2)	Oral and maxillofacial surgeon	Prosthodontist	2.181	3.585	1.000
		Periodontist	8.525	3.585	0.112
		Dentist	10.440^*∗*^	3.585	0.025^*∗*^
				
	Prosthodontist	Oral and maxillofacial surgeon	−2.181	3.585	1.000
		Periodontist	6.344	3.585	0.473
		Dentist	8.259	3.585	0.135
				
	Periodontist	Oral and maxillofacial surgeon	−8.525	3.585	0.112
		Prosthodontist	−6.344	3.585	0.473
		Dentist	1.915	3.585	1.000
				
	Dentist	Oral and maxillofacial surgeon	−10.440^*∗*^	3.585	0.025^*∗*^
		Prosthodontist	−8.259	3.585	0.135
		Periodontist	−1.915	3.585	1.000

Torsional moment (Ch3)	Control	Oral and maxillofacial surgeon	16.215^*∗*^	0.928	<0.001^*∗*^
		Prosthodontist	14.999^*∗*^	0.928	<0.001^*∗*^
		Periodontist	17.047^*∗*^	0.928	<0.001^*∗*^
		Dentist	18.704^*∗*^	0.928	<0.001^*∗*^
				
	Oral and maxillofacial surgeon	Control	−16.215^*∗*^	0.928	<0.001^*∗*^
		Prosthodontist	−1.216	0.928	1.000
		Periodontist	0.832	0.928	1.000
		Dentist	2.490	0.928	0.079
				
	Prosthodontist	Control	−14.999^*∗*^	0.928	<0.001^*∗*^
		Oral and maxillofacial surgeon	1.216	0.928	1.000
		Periodontist	2.048	0.928	0.285
		Dentist	3.705^*∗*^	0.928	0.001^*∗*^
				
	Periodontist	Control	−17.047^*∗*^	0.928	<0.001^*∗*^
		Oral and maxillofacial surgeon	−0.832	0.928	1.000
		Prosthodontist	−2.048	0.928	0.285
		Dentist	1.658	0.928	0.755
				
	Dentist	Control	−18.704^*∗*^	0.928	<0.001^*∗*^
		Oral and maxillofacial surgeon	−2.490	0.928	0.079
		Prosthodontist	−3.705^*∗*^	0.928	<0.001^*∗*^
		Periodontist	−1.658	0.928	0.755

*∗* refers to <0.05.

**Table 3:** Mean bending and torsional moment values according to specialty.

Moment type	Specialty	Mean (Ncm)
Bending moment (Ch1)	Oral and maxillofacial surgeon	22.868
	Prosthodontist	29.420
	Periodontist	23.075
	Dentist	17.546

Bending moment (Ch1)	Oral and maxillofacial surgeon	22.917
	Prosthodontist	20.736
	Periodontist	14.392
	Dentist	12.476

Torsional moment (Ch3)	Control	25.988
	Oral and maxillofacial surgeon	9.773
	Prosthodontist	10.989
	Periodontist	8.941
	Dentist	7.283

**Table 4:** *t*-test for equality of means according to gender and bending moment.

Moment type	*t*	Two-tailed (*p*)
Bending moment (Ch1)	−2.336	0.021^*∗*^
Bending moment (Ch2)	−1.494	0.137

*∗* refers to <0.05.

**Table 5:** ANOVA for Ch3 torsional moment according to gender.

Moment type	*F*	Sig.
Torsional moment (Ch3)	259.502	<0.001^*∗*^

*∗* refers to <0.05.

**Table 6:** Bonferroni test for Ch3 torsional moment according to gender.

Moment type	(*I*) Gender	(*J*) Specialty	Mean difference (*I* − *J*) (Ncm)	Std. error	Sig.
Torsional moment (Ch3)	Female	Male	−2.096^*∗*^	0.664	0.006^*∗*^
		Control	−17.789^*∗*^	0.813	<0.001^*∗*^
				
	Male	Female	2.096^*∗*^	0.664	0.006^*∗*^
		Control	−15.693^*∗*^	0.813	<0.001^*∗*^
				
	Control	Female	17.789^*∗*^	0.664	<0.001^*∗*^
		Male	15.693^*∗*^	0.813	<0.001^*∗*^

*∗* refers to <0.05.

**Table 7:** Mean bending and torsional moment values according to gender.

Moment type	Gender	Mean (Ncm)
Bending moment (Ch1)	Female	19.718
	Male	26.737

Bending moment (Ch2)	Female	15.693
	Male	19.567

Torsional moment (Ch3)	Female	8.199
	Male	10.295
	Control	25.988

**Table 8:** Evaluation of the correlation between initial tightening torque and gap/fraction on the loading side using Spearman's rho test.

Correlation coefficient	Starting	Case
Spearman's Rho		
Starting		
Correlation coefficient	1.000	−0.001
Two-tailed (*p*)	—	0.996
*N*	20	20
Case		
Correlation coefficient	−0.001	1.000
Two-tailed (*p*)	0.996	—
*N*	20	20

**Table 9:** Complications of implants undergoing dynamic loading testing.

Implant	Experimental group			Control group	
	Fractures	Opening	Loosening	Fractures	Loosening
3.7 mm + standard	3	2	0	1	4
3.7 mm + solid	4	1	0	1	4
4.1 mm + standard	2	1	2	1	4
4.1 mm + solid	0	4	1	0	5
Total	9	8	3	3	17
